# Crosstalk between cholesterol metabolism and psoriatic inflammation

**DOI:** 10.3389/fimmu.2023.1124786

**Published:** 2023-05-10

**Authors:** Lingling Luo, Youming Guo, Lihao Chen, Jing Zhu, Chengrang Li

**Affiliations:** ^1^ Department of Dermatology, Hospital for Skin Disease, Institute of Dermatology, Chinese Academy of Medical Sciences and Peking Union Medical College, Nanjing, Jiangsu, China; ^2^ Department of Dermatology, Jiangsu Key Laboratory of Molecular Biology for Skin Diseases and Sexually Transmitted Infections, Nanjing, Jiangsu, China

**Keywords:** cholesterol metabolism, immunity, inflammation, immunometabolism, psoriatic inflammation

## Abstract

Psoriasis is a chronic autoinflammatory skin disease associated with multiple comorbidities, with a prevalence ranging from 2 to 3% in the general population. Decades of preclinical and clinical studies have revealed that alterations in cholesterol and lipid metabolism are strongly associated with psoriasis. Cytokines (tumor necrosis factor-α (TNF-α), interleukin (IL)-17), which are important in the pathogenesis of psoriasis, have been shown to affect cholesterol and lipid metabolism. Cholesterol metabolites and metabolic enzymes, on the other hand, influence not only the biofunction of keratinocytes (a primary type of cell in the epidermis) in psoriasis, but also the immune response and inflammation. However, the relationship between cholesterol metabolism and psoriasis has not been thoroughly reviewed. This review mainly focuses on cholesterol metabolism disturbances in psoriasis and their crosstalk with psoriatic inflammation.

## 1 Introduction

Psoriasis is a prevalent, persistent, inflammatory skin condition. Psoriasis is estimated to affect approximately 125 million people worldwide (1), with an incidence rate ranging from 30.3 to 321.0 cases per 100,000 person-years (2, 3) and a population prevalence ranging from 0.51% to 11.43% in adults and 0% to 1.37% in children (4). Psoriasis is now widely recognized as a systemic inflammatory disease rather than a merely cutaneous disease. In patients with psoriasis, systemic comorbidities such as cardiovascular diseases, metabolic syndrome, gastrointestinal diseases (inflammatory bowel disease, liver diseases), chronic kidney diseases, malignancy, infection, psychiatric disorders, psoriatic arthritis, and sleep apnea have been observed (5, 6). And the incidence of these comorbidities rises in direct proportion to the severity and duration of psoriasis (7). Moreover, a study found that comorbidities of cardiovascular diseases, diabetes, chronic obstructive pulmonary disease, cancer, chronic kidney disease, and stroke respectively increase the mortality rate of patients with psoriasis by 15.5%, 5.9%, 8.7%, 11.7%, 4.2%, and 4.7% (8). The majority of current research on the comorbidities of psoriasis mainly focuses on cardiovascular diseases and metabolic syndrome, both of which are intimately connected to lipid metabolism (9).

Lipids play critical roles in maintaining internal homeostasis as components of cell membranes and hormone components, a form of energy storage, and mediators of cellular signaling (10). The LIPID MAPS Consortium developed a “Comprehensive Classification System for Lipids” that divides lipids into eight groups based on their chemical and functional properties; These groups include fatty acyls, glycerolipids, glycerophospholipids, sphingolipids, and sterol lipids including cholesterol, prenol lipids, saccharolipids, and polyketides (11). Cholesterol metabolism, which is a critical metabolic bioprocess *in vivo*, has been confirmed to interact with the immune system (12). The link between cholesterol metabolism and immunological response and inflammation has been a “hotspot” for research since the millennium, giving rise to the concept of “immunometabolism” (13) Because of the link between cholesterol metabolism and the immune response and inflammation, it is common to find disturbances of cholesterol metabolism in chronic inflammatory diseases (12).

Psoriasis is a typical inflammatory disease. In psoriasis, the activation of immune cells (dendritic cells, T helper 1 (Th1) cells, and T helper 17 (Th17) cells, keratinocytes (KCs), macrophages, and neutrophils) and overproduction of key inflammatory cytokines (interferon (IFN)-α, IFN-γ, tumor necrosis factor (TNF)-α, interleukin (IL)-1β, IL-6, IL-17, IL-22, IL-12/IL-23, chemokines and antimicrobial peptides) are responsible for the chronic inflammation and epidermal hyperproliferation and impaired differentiation (14). Research on cholesterol metabolism in psoriasis began in the 20th century (15). The early research mostly focused on the serum cholesterol level in psoriasis patients, but the results were contradictory (16). Following that, a plethora of studies confirmed the presence of abnormalities in cholesterol metabolism in psoriasis patients. In a review published in 2010, Aldona Pietrzak et al. summarized studies on lipids in psoriasis and found that patients with psoriasis have a significant increase in the serum level of total cholesterol, low-density lipoprotein (LDL) cholesterol, triglycerides, and very low-density lipoprotein (VLDL) cholesterol, and a significant decrease in the serum level of high-density lipoprotein (HDL) cholesterol, compared to healthy individuals (17). Furthermore, distortions in cholesterol metabolism might hasten the development of autoimmune illnesses (e.g., psoriasis, rheumatoid arthritis (RA), and systemic lupus erythematosus) by altering the phenotype and function of innate immune cells (e.g., macrophages, dendritic cells) and adaptive immune cells (e.g., T cells and B cells) (12, 18, 19). Similarly, cytokine networks in psoriatic circulation can affect lipoprotein particle size, concentration, and function, impairing cholesterol transport and efflux (20). Hence, cytokine networks involved in cholesterol metabolism and inflammation may be the primary mechanism linking psoriasis to cardiovascular comorbidities (20). Disruptions in lipid metabolism may also impair the function of KCs, contributing to the development of psoriasis (21).

Previous research has mostly focused on the alteration of serum lipid and epidermal phospholipid metabolism in psoriatic patients. In recent years, research on cholesterol metabolism in psoriasis has made significant strides. Psoriatic inflammation also affected certain cholesterol intermediates, enzymes, and metabolites. In this review, we mainly discuss the alterations and interactions between psoriatic inflammation and molecules related to cholesterol metabolism.

## 2 Cholesterol metabolism

In the following section, we briefly discussed the process of cholesterol metabolism in the small intestine, serum, and cells.

### 2.1 Absorption of cholesterol in the small intestine

**Figure 1:**
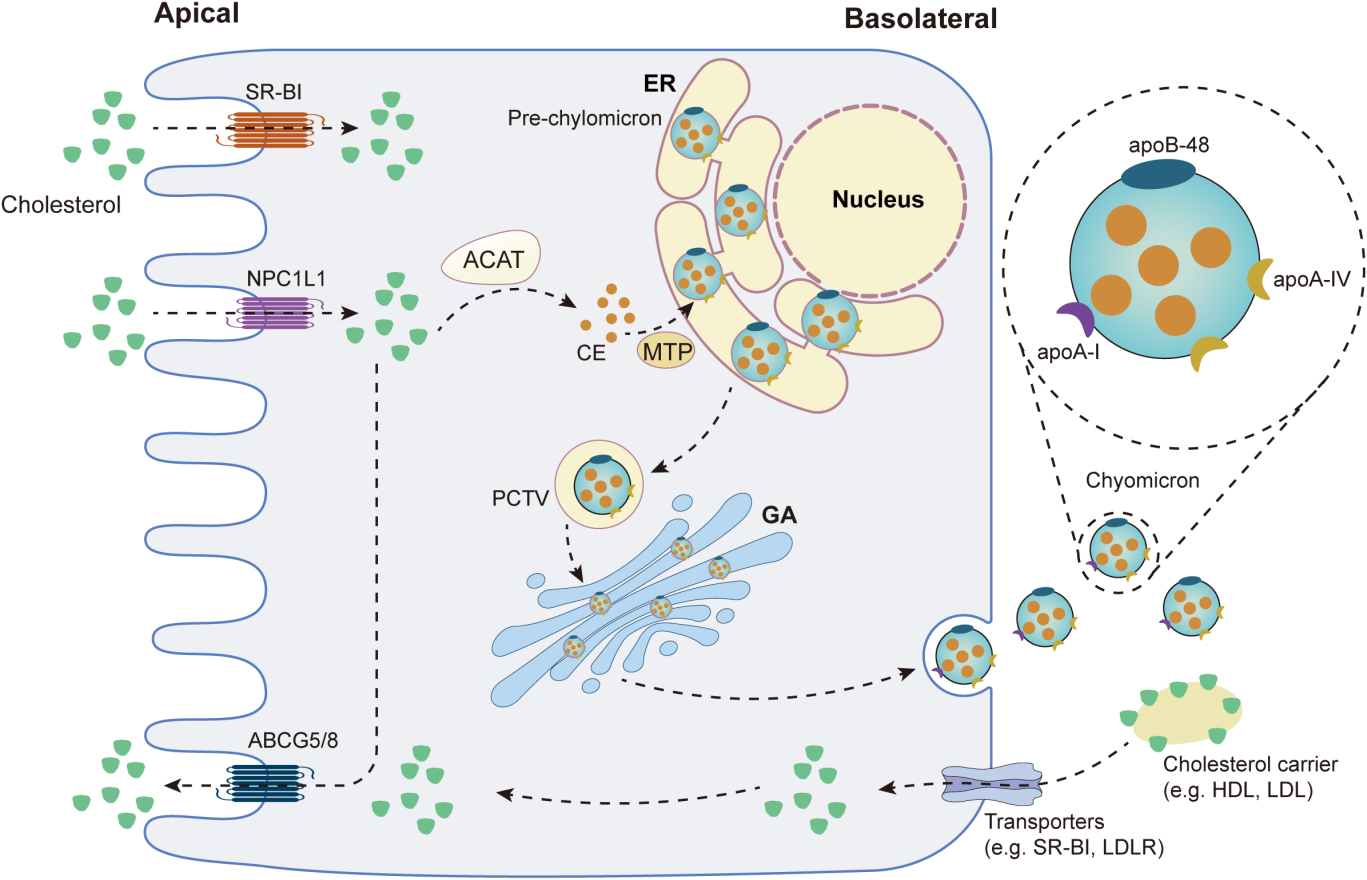
Cholesterol metabolism in the small intestine. SR-B1: scavenger receptor type 1; NPC1L1: Niemann-Pick C1-Like 1; ABCG5/8: ATP Binding Cassette Subfamily G Member 5/Member 8; ACAT: acyl-CoA:cholesterol acyltransferase; CE: cholesteryl ester; MTP: Microsomal triglyceride transfer protein; ER: endoplasmic reticulum; PCTV: pro-chylomicron transport vesicle; GA: golgi apparatus; HDL: high-density lipoprotein-cholesterol; LDL: low-density lipoprotein-cholesterol; LDLR: low-density lipoprotein receptor.

Cholesterol binds to bile acid micelles in the small intestine and is transported to the brush border of enterocytes, where it binds to Niemann-Pick C1-Like 1 (NPC1L1) and is delivered to the endocytic cycle compartment by clathrin-mediated endocytosis (22). NPC1L1 can be recycled back to the plasma membrane with the help of the LIM domain and actin-binding protein 1 (LIMA1), cell division cycle 42 (CDC42), the motor protein myosin Vb and actin filaments (23). Acyl-CoA:cholesterol acyltransferase (ACAT) then converts free cholesterol absorbed by enterocytes into cholesteryl esters (CEs), which are then incorporated into chylomicrons (CM), a triglyceride-rich lipoprotein type with a CEs-rich core and a single molecule of apolipoprotein B (ApoB)-48 on its surface (22). Microsomal triglyceride transfer protein (MTP) and apolipoprotein A (ApoA)-IV are essential for chylomicron assembly and size regulation (24). The endoplasmic reticulum (ER) then generates a pro-chylomicron transport vesicle (PCTV) to carry pro-chylomicrons to the Golgi apparatus (24). PCTV was translocated to the Golgi *via* the soluble N-ethylmaleimide-sensitive factor attachment protein receptor (SNARE) complex (24). Pro-chylomicrons mature into mature CM after the addition of Apo-I and enzymatic modification in the Golgi apparatus, after which they are transported to the basolateral membrane and released into the intercellular space by exocytosis (24). After entering the lamina propria from the intercellular space, CM enters the bloodstream *via* the thoracic duct or enters the liver through the portal vein (22). **Figure 1** summarizes cholesterol absorption in the small intestine.

### 2.2 The biosynthesis of cholesterol

**Figure 2:**
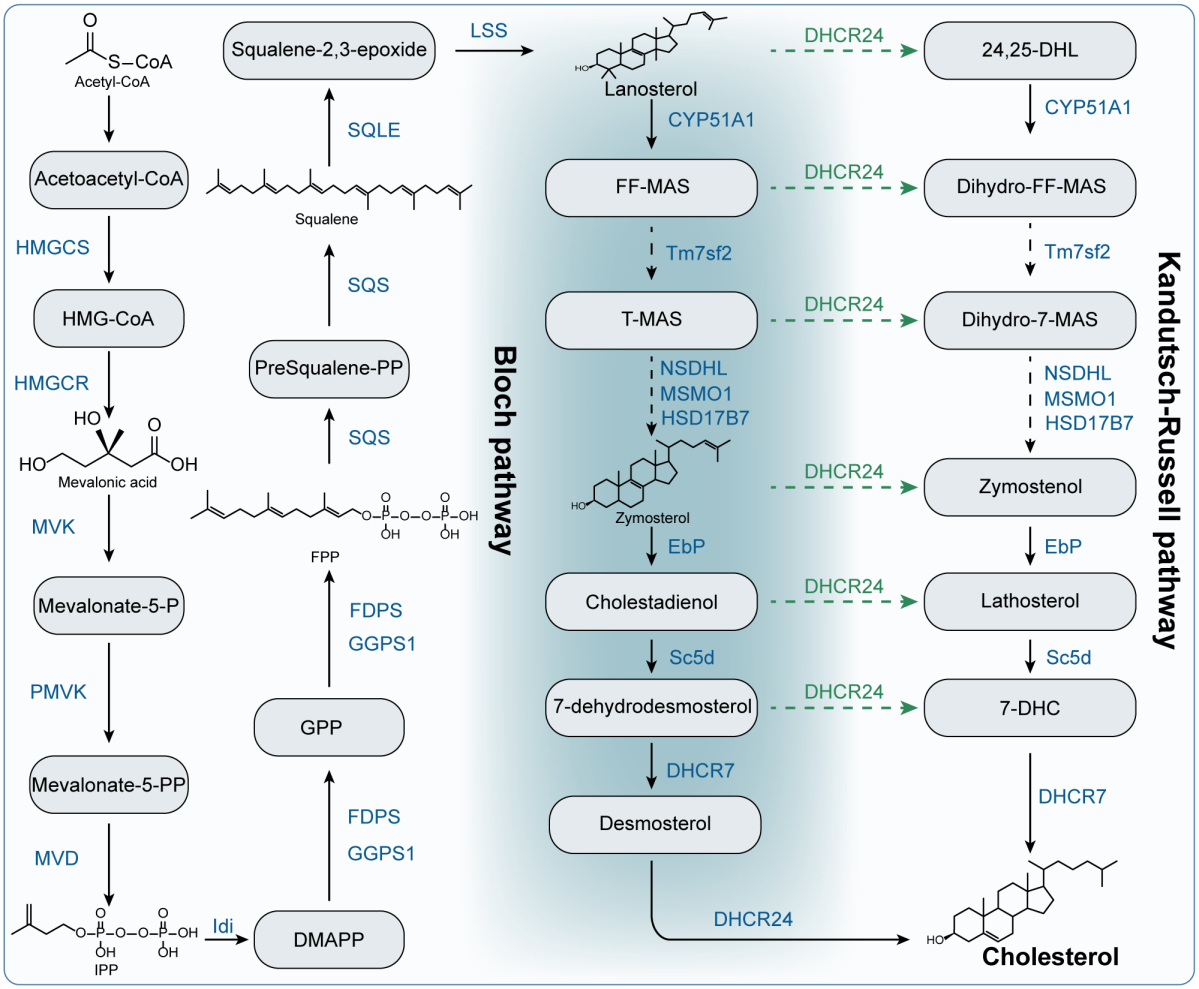
The biosynthesis of cholesterol. HMGCS: Hydroxymethylglutaryl-CoA synthase; HMG-CoA: 3-hydroxy-3-methyl–CoA. HMGCR: 3-Hydroxy-3-Methylglutaryl-CoA Reductase; MVK: mevalonate kinase; Mevalonate-5-P: mevalonate-5-phosphate; PMVK: phosphomevalonate kinase; Mevalonate-5-PP: mevalonate-5-pyrophosphate; MVD: mevalonate diphosphate decarboxylase; IPP: isopentenyl pyrophosphate; Idi: isopentenyl diphosphate isomerase; DMAPP: dimethylallyl diphosphate; FDPS: farnesyl diphosphate synthase; FPP: farnesyl pyrophosphate; GPP: geranyl pyrophosphate; GGPS1: geranylgeranyl diphosphate synthase 1; SQS: squalene synthase; presqualene-PP: presqualene pyrophosphate; SQLE: squalene epoxidase; LSS: lanosterol synthase; CYP51A1: Cytochrome P450 Family 51 Subfamily A Member 1; FF-MAS: Follicular fluid meiosis-activating sterol. T-MAS: testis meiosis-activating sterol. Tm7sf2: transmembrane 7 superfamily member 2; MSMO1: methylsterol monooxygenase 1; NSDHL: NAD(P) Dependent Steroid Dehydrogenase-Like; HSD17B7: Hydroxysteroid 17-Beta Dehydrogenase 7; EbP: emopamil binding protein; Sc5d: sterol C5-desaturase; DHCR7: 7-dehydrocholesterol reductase; DHCR24: 24-dehydrocholesterol reductase; 24,25-DHL: 24,25-Dihydrolanosterol; 7-DHC: 7-dehydrocholesterol.

The liver is the principal organ for cholesterol synthesis, producing almost 50% of all generated cholesterol. In addition, almost all cells in the body can produce cholesterol (25). The synthesis of cholesterol is a complicated process with an expensive energy cost (23). In this section, we outlined the key metabolites and metabolic enzymes and the process of cholesterol synthesis in **Figure 2**. Cholesterol is made and then release into the bloodstream as VLDL particles that is covered with ApoB100 (22).

### 2.3 Cholesterol transport and lipoproteins metabolism

**Figure 3:**
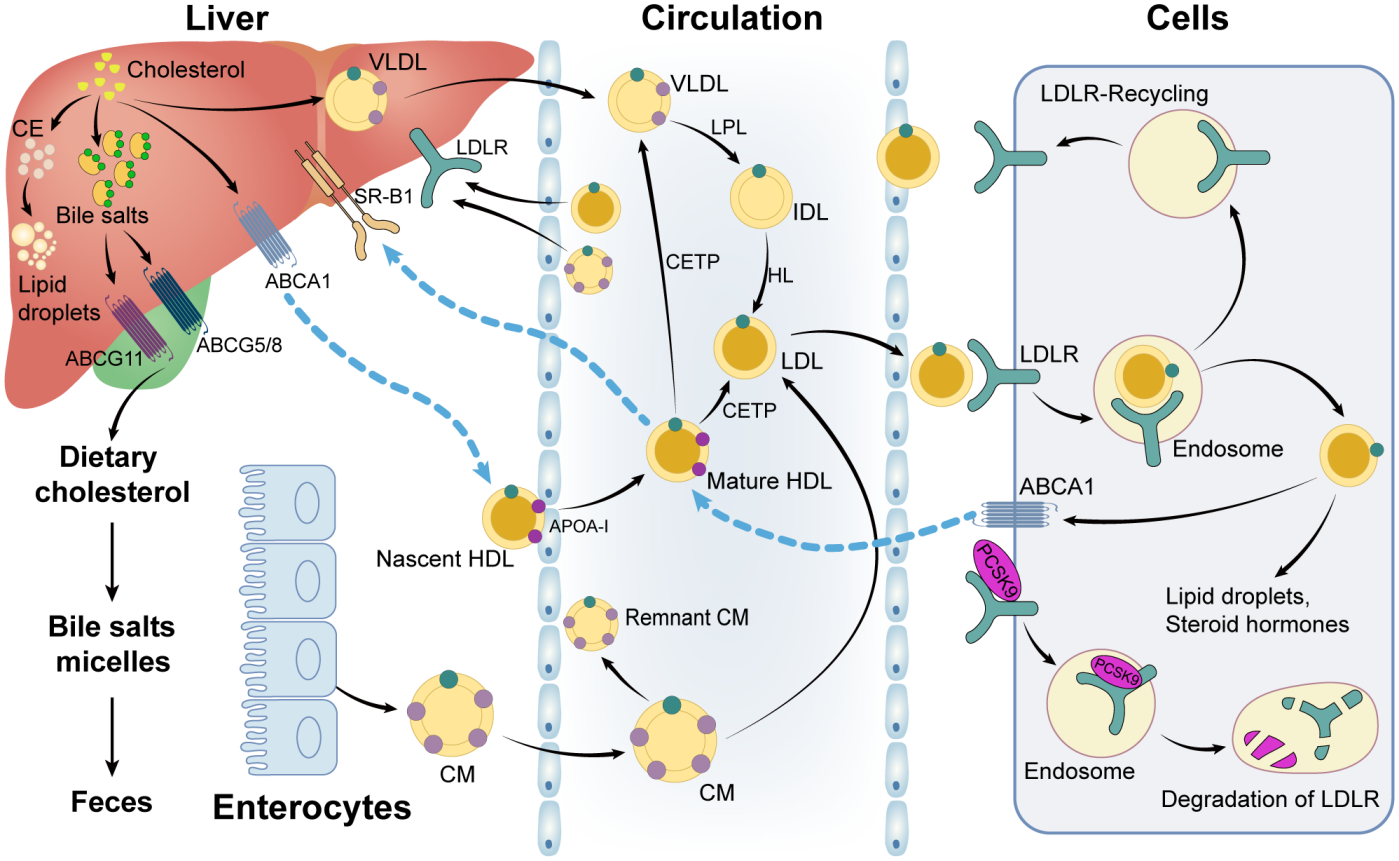
Cholesterol and lipoprotein metabolism. CE: cholesteryl ester; VLDL: very low-density lipoprotein-cholesterol; ABCG11: ATP Binding Cassette Subfamily G Member 11; ABCG5/8: ATP Binding Cassette Subfamily G Member 5/Member 8; SR-B1: scavenger receptor type 1; ABCA1: ATP Binding Cassette Subfamily A Member 1; LDLR: low-density lipoprotein receptor; LPL: lipoprotein lipase; IDL: intermediate-density lipoprotein; HL: hepatic lipase; HDL: high-density lipoprotein-cholesterol; LDL: low-density lipoprotein-cholesterol; CETP: cholesteryl ester transfer protein; CM: chylomicron; PCSK9: proprotein convertase subtilisin/kexin type 9; ApoA-1: apolipoprotein A 1.

Once CM and VLDL enter the circulation, the triglycerides in the core are digested into chylomicron fragments and LDL cholesterol by lipoprotein lipase (LPL) (22, 26). VLDL is hydrolyzed by LPL into intermediate-density lipoprotein (IDL), which is then converted into LDL *via* the hydrolysis of hepatic lipase (HL) (22). In addition, the triglycerides, CEs, and phospholipids in the HDL particles can be enriched to form CM and VLDL particles in the circulation by cholesteryl ester transfer proteins (CETP) and phospholipid transfer proteins (PLTP), which are accompanied by the interchange of apolipoproteins on the surface of lipoprotein particles (22, 27). Hereafter, chylomeric fragments and LDL particles are taken up by the low-density lipoprotein receptor (LDLR) and low-density lipoprotein receptor-related protein 1 (LRP1) in cells, while HDL particles are bound to the scavenger receptor type 1 (SR-B1) (26, 28). CEs are selectively delivered into cells when HDL particles attach to scavenger receptor type 1 (SR-B1) on the surface of hepatocytes. Following that, hormone-sensitive lipase (HSL) hydrolyzes CEs to liberate cholesterol (26). The processes of cholesterol transport and lipoprotein metabolism are shown in **Figure 3**.

### 2.4 Cellular utilization, storage and efflux of cholesterol

After being taken up by cells, the CEs carried by LDL particles are digested by lysosomal acid lipase to release free cholesterol, which is subsequently co-transferred to the lysosomal membrane by NPC intracellular cholesterol transporter 1 (NPC1), NPC2, and lysosome-associated membrane protein 2(LAMP2) and transported to other cell compartments (mainly ER, re-esterified to form CE) (23). HDL binds to SR-B1 on hepatocytes, delivering CEs from lipoproteins to the liver (23). Absorbed cholesterol in cells can be utilized to act as a component of biological membranes or to synthesize oxysterols (28). And in gonadal tissue, adrenal glands and brain cells, cholesterol can be used for steroid hormones (28). In the liver, most sterols are further metabolized into bile acids and bile salts, which are released into extracellular space through ATP Binding Cassette Subfamily B Member 11 (ABCB11) transporters to produce bile salt-phosphatidylcholine micelles for excretion (22). The metabolic processes of oxysterols, bile acids, and steroid hormones, as well as their roles in immune regulation, have been well described (29–32), and we will not go over them here.

While all cells can synthesize cholesterol, the vast majority are unable to catabolize it, forcing excess cholesterol to be excreted or stored in cytoplasmic lipid droplets (23). ACAT is in charge of esterifying free cholesterol to generate CEs that are stored in lipid droplets; the ATP-binding cassette (ABC) transporter superfamily is in charge of the efflux of intracellular cholesterol (23, 28). The ATP Binding Cassette Subfamily G Member 1 (ABCG1) is mainly expressed in macrophages and frequently dimerizes with another ABCG1 or ABCG4 to form a functional transport protein that primarily mediates cholesterol efflux to HDL, albumin, or other lipoproteins (23). ATP Binding Cassette Subfamily A Member 1 (ABCA1) is a ubiquitously expressed transporter that mediates cholesterol and phospholipid efflux to apoA-I, resulting in HDL formation. The ATP Binding Cassette Subfamily G Member 5 (ABCG5)/ATP Binding Cassette Subfamily G Member 8 (ABCG8) complex is primarily expressed in enterocyte brush boundaries and hepatocyte tubular membranes, where it mediates direct transintestinal cholesterol excretion (TICE) into the intestinal lumen or hepatic sterol efflux into bile (23, 24). The processes of cellular utilization, storage and efflux of cholesterol were summarized in **Figure 3**.

## 3 Cholesterol metabolism and psoriatic inflammation

In this part, we will look at how molecules, enzymes, and intermediates involved in cholesterol metabolism affect psoriasis and/or psoriatic inflammation.

### 3.1 Intestinal absorption of cholesterol: NPC1L1

Except for the intestine, NPC1L1 is also expressed in the liver, where it regulates hepatobiliary cholesterol excretion by transporting cholesterol in the bile to hepatocytes (33). NPC1L1 has been demonstrated to regulate inflammatory responses *in vivo*. NPC1L1 inhibition has been shown to reduce the production of pro-inflammatory proteins like β-catenin and p- extracellular signal-regulated kinase (ERK), cell proliferation and the inflammatory response in the intestine of mice (34). Ezetimibe, an NPC1L1-targeting inhibitor, can reduce the absorption of dietary and bile cholesterol, hence lowering lipid levels. Ezetimibe has been proven to effectively cut diet-induced circulating cholesterol levels while also inhibiting hepatic lipid build-up and lowering inflammatory factors such as high-sensitivity C-reactive protein (hs-CRP), monocyte chemoattractant protein-1, IL-1β, IL-6, TNF-α, and oxidative stress (35–37). Experiments on the macrophage model demonstrated that ezetimibe could lead to an anti-inflammatory response by decreasing the transcription of nuclear factor kappa-light-chain-enhancer of activated B cells (NF-κB) and blocking the NLR Family Pyrin Domain Containing 3 (NLRP3) inflammasome- IL-1β pathway (38). Ezetimibe can also reduce oxidative stress and inflammatory responses mediated by caspase-1 and IL-1β through the AMP-activated protein kinase (AMPK)/NF-E2–related factor 2 (Nrf2)/thioredoxin interacting protein (TXNIP) pathway (39). And ezetimibe reduces monocyte chemoattractant protein 1-induced monocyte migration (40). Furthermore, ezetimibe was found to lower the frequency of Th17 and Th1 cells in RA mice and humans, as well as suppress the differentiation of Th17 cells and the production of pro-inflammatory molecules such as IL-1, IL-17, IFN-γ, TNF-α, and IL-6, thereby slowing the progression of RA (41). It has been documented that NPC1L1 could be affected by immune cells. Propionate produced by the gut microbiota suppressed intestinal NPC1L1 expression and corrected hyperlipidemia by increasing regulatory T-cell numbers and IL-10 (42). There is a scarcity of studies on ezetimibe in psoriasis. Only a clinical study revealed that ezetimibe was not linked to the risk of psoriasis (point estimates for ezetimibe revealed decreased psoriasis risk, but the null is included in the confidence intervals) (43). However, on the basis of the impacts of NPC1L1 on lipid metabolism, cell proliferation, and inflammation, we still hypothesize that inhibiting NPC1A1 may help relieve the symptoms of psoriasis, but further studies are needed to validate this hypothesis.

### 3.2 Cholesterol synthase and intermediates

#### 3.2.1 HMG-CoA reductase in mevalonate pathway

Statins, a commonly used lipid-lowering drug, reduce cholesterol by inhibiting the enzyme 3-hydroxy-3-methylglutaryl coenzyme A (HMG-CoA) reductase. And they are widely used to treat hyperlipidemia and cardiovascular diseases. In addition to lowering cholesterol levels, statins also modulate the immune system. Statins impede antigen presentation and lymphocyte activation by inhibiting the production and interaction of intercellular adhesion molecule 1 (ICAM-1), lymphocyte function-associated antigen 1 (LFA-1), and the major histocompatibility complex, class II (MHC-II) (44). And LFA-1 is a target of efalizumab, which has been shown to be effective in psoriatic treatment (45). Moreover, statins can significantly downregulate the expression of Th1 chemokine receptors C-C Motif chemokine receptor (CCR)-5 and C-X-C chemokine receptor type (CXCR)-3 on T cells, block the release of chemokines from endothelial cells, reduce the proliferation of monocytes, and inhibit the activation of natural killer (NK) cells and leukocytes (44). *In vitro*, statins inhibit the release of inducible nitric oxide synthase (iNOS) and pro-inflammatory cytokines such as TNF-α, IFN-γ, IL-8, IL-1, IL-17, and IL-6 (46, 47). Statins also inhibit the Th17 cells and the activation and migration of Th1 cells (48). Statins regulate the proliferation of KCs. Pravastatin inhibits KCs proliferation by modulating cell cycle-related proteins *via* the protein kinase B (AKT) and ERK pathways (49). And *in vitro* experiments conducted on human keratinocytes cells (HaCaT) found that pitavastatin and simvastatin can inhibit not only keratinocyte proliferation but also the release of vascular endothelial growth factor (VEGF) from KCs (50, 51). All these studies implied the potential therapeutic role of statins in psoriasis. Consistent with the *in vitro* studies, animal experiments showed that atorvastatin dramatically improved the clinical symptoms and histological characteristics, and decreased the release of IL-17A, IL-6 and TNF-α, and NF-κB activation in psoriatic mouse models (52).

Nonetheless, statins have an uneven impact on psoriasis. Varieties of clinical research confirm the therapeutic benefit of statins in psoriasis (53–58). In a double-blind trial, 30 people with plaque psoriasis were randomly put into one of two groups: the simvastatin therapy group (receiving a therapy of oral simvastatin (40 mg/d) plus topical steroids (Vaseline with 50% betamethasone)) or the control group (receiving a therapy of placebo plus same topical steroids). After 8 weeks of treatment, the psoriasis area severity index (PASI) score of both groups decreased considerably, but the decrease was greater in the simvastatin therapy group (59). And the combination of oral atorvastatin and topical betamethasone could reduce the PASI score, hs-CRP levels, and cardiovascular risk of patients with mild to moderate plaque psoriasis (60). However, some studies suggest that oral statins do not affect the progression of psoriasis. Mendelian randomization studies revealed that statins were not linked to a reduced risk of psoriasis (43, 61). According to a study by Brauchli et al., long-term statin therapy failed to lower the risk of new-onset psoriasis (62). Furthermore, additional oral atorvastatin or simvastatin was not associated with a therapeutic benefit in psoriasis patients receiving standard topical therapy or narrowband UVB therapy (63–65). Another study evaluated the efficacy of adding topical simvastatin to topical calcipotriol in psoriasis. Even though the PASI scores kept going down in both groups, topical simvastatin wasn’t statistically better at treating psoriasis (66). Other studies have found that statins may worsen psoriasis lesions (67, 68). A meta-analysis of randomized clinical studies in 2019 and a systematic evaluation in 2022 revealed that oral and topical statins could significantly improve psoriatic lesions and lower the PASI score, especially simvastatin (69, 70).

In addition, statins have been shown to reduce vascular inflammation and the risk of developing cardiovascular disease and chronic kidney disease in psoriasis patients (71–73).

Overall, statins are still thought to have beneficial therapeutic effects in psoriasis. However, psoriatic individuals are not recommended to receive statins commonly; only those with elevated cholesterol levels, diabetes, or an intermediate or high risk of developing cardiovascular disease should be given statins (74).

#### 3.2.2 Mevalonate pathway (HMG-CoA, mevalonate kinase, phosphomevalonate kinase, mevalonate diphosphate decarboxylase and isoprenoids)

The enzymes mevalonate kinase (MVK), phosphomevalonate kinase (PMVK), and mevalonate diphosphate decarboxylase (MVD) are crucial synthetases in the mevalonate pathway for the synthesis of isopentenyl pyrophosphate (IPP). The loss-of-function mutations in these genes may lead to porokeratosis, which is characterized by an incomplete differentiation and apoptosis of KCs (75–77) and is an “autoinflammatory keratosis” with a gene expression profile similar to psoriasis (78, 79). Mutations in the MVK and PMVK genes are the cause of mevalonate kinase deficiency, which is a disease characterized by recurrent fever and high levels of TNF-α and IL-6 (80, 81). MVK deficiency can result in mitochondrial malfunction and autophagy, as well as activating the NLR family pyrin domain containing 3 (NALP3) inflammasome, which causes IL-1 over-secretion and downstream inflammatory activation (80). Besides, dysregulation of IL-1β, IL-5, IL-6, IL-9, IL-17, granulocyte colony-stimulating factor, monocyte chemotactic protein-1, TNF-α, and IL-4 were observed in mevalonate kinase deficiency patients and mice (82). Furthermore, MVK plays a vital function in both trained immunity and inflammatory propensity (83). Although these three enzymes are proven to affect the function of KCs and psoriasis-related cytokines, there has been no research on the involvement of MVK, PMVK, and MVD in psoriasis.

Currently, it is thought that the effects of HMG-CoA reductase and the MVK on inflammation and cell proliferation may be mediated by affecting isoprenoids during the cholesterol synthesis process (44, 80).

IPP, farnesyl pyrophosphate (FPP), and geranylgeranyl pyrophosphate (GGPP) belong to isoprenoids. FPP and GGPP regulate the prenylation of the most notable superfamily of small GTPases (Ras, Rab, Rho, and Rac), which activate downstream signaling pathways and regulate cellular function and inflammatory activities (84). Isoprenoids have an impact on both the innate and adaptive immune systems. When inhibiting the GGTase I of macrophages, lipopolysaccharides (LPS) made them secrete more IL-1 and TNF by making Rac1, RhoA, and Cdc42 bind more GTP. Blocking Rac1 turned this effect around (85). Inhibiting GGTase decreased the synthesis of GGPP, further suppressing the toll-like receptor (TLR)9- phosphoinositide 3-kinases (PI3K)-AKT- glycogen synthase kinase-3 (GSK3) axis-induced IL-10 production in B cells and dampening Th1 responses (85). Furthermore, inhibiting farnesyl diphosphate synthase (FDPS) decreases FPP levels, which reduces Th1 cytokines release such as IL6, IL-1β, and TNF-αwhile increasing Th2 responses (86). IPP has been shown to increase the expression of the IL-9 receptor and the IL-23 receptor on Vγ9Vδ2 T cells in patients with psoriatic arthritis (PA), which increases the responsiveness of Vγ9Vδ2 T cells to IL-23 and IL-9 and dramatically promotes the expression of IL-17 and IFN-γ (87).

#### 3.2.3 The synthesis of lanosterol from squalene (squalene synthase, squalene epoxidase, lanosterol synthase)

In addition to lowering cholesterol and preventing atherosclerosis, squalene has also been shown to reduce inflammation. It does this by increasing the production of anti-inflammatory molecules like IL-10, IL-4, and IL-13 and by directing macrophages toward the M2 phenotype (88, 89). In LPS-stimulated RAW264.7 murine macrophages, squalene dramatically decreased nitric oxide (NO) production and sequestration of TNF-α and C-C motif chemokine ligand 2 (CCL2) (89). Due to the six double bonds in squalene, light can oxidize squalene to produce squalene peroxide (90).. Treatment of HaCaT with squalene peroxide could promote cell proliferation and lysyl oxidase (LOX) activity, an enzyme involved in lipid peroxide synthesis, the product of which is believed to be involved in the development of inflammatory skin diseases with excessive proliferation of KCs, such as psoriasis (90). Squalene peroxide also dramatically increased NF-κB nuclear translocation, which was followed by increased expression and secretion of the pro-inflammatory cytokine IL-6, as well as elevated peroxisome proliferator-activated receptors (PPARs) mRNA and protein levels, which takes an important part in the pathogenesis of psoriasis (90).

Squalene synthase (SQS) is an enzyme responsible for catalyzing the production of squalene from FPP. In clinical trials, it was shown that inhibitors targeting SQS are efficient at lowering both cholesterol and C-reactive protein levels (91). It is speculated that SQS may be involved in the development of psoriasis. On one hand, SQS stimulates cell proliferation by raising the cholesterol levels in lipid rafts and activating the NF-κB or AKT pathway (92, 93). On the other hand, SQS could regulate the innate immune process and inflammation *via* isoprenoids. Small interfering RNAs (SiRNAs) targeting SQS suppress bacterial and LPS-mediated innate immune responses, resulting in a decrease in IL-6 and chemokine (C-X-C motif) ligand (CXCL)-8 production, which is consistent with the effects of FPP and GGPP stimulation (94). And, SQS inhibitor lapaquistat acetate (TAK-475) could lower the elevated IL-6, and TNF-α caused by isoprenoids (GGPP and FPP, primary GGPP) in patients with mevalonate kinase deficiency (80, 95). Furthermore, SQS overexpression may increase the expression of TNF-αreceptors in cells, resulting in a stronger cellular response to TNF-α (96). However, another study also found that inhibiting SQS may impair Fc receptor-mediated phagocytosis in macrophages by affecting cholesterol production rather than isoprenoid (97). Positive feedback may exist between pro-inflammatory factors and squalene synthase, as TNF-α and IL-1 could promote the expression of squalene synthase in mouse liver (98). However, there are no reports on the expression level of SQS and the application of lipid-lowering drugs targeting SQS in psoriasis.

Squalene epoxidase (SQLE), also known as squalene monooxygenase, catalyzes squalene epoxidation to create 2,3-oxidized squalene, and its elimination can lead to an accumulation of squalene. According to a study by Xiaoqing Xu et al., increased activator protein 1(AP-1) in psoriasis may promote the onset of hyperkeratosis, parakeratosis, acanthosis, and immunological abnormalities by targeting SQLE (99). There has been limited research on SQLE and inflammation. A bioinformatic analysis showed that SQLE promotes cell proliferation, as well as being associated with immune cell infiltration and the immune microenvironment (100).

Excessive proliferation and defective differentiation of KCs are typical characteristics of psoriasis, and the mutations in the lanosterol synthase (LSS) gene have been proven to induce over-proliferation and differentiation disorders of KCs (101). Research on the relationship between LSS and inflammation is scarce. Only McCrae et al. found that LSS inhibition may increase the production of 24(S),25 epoxycholesterol, resulting in an increase in the antiviral protein IFN-βand regulating innate antiviral immunity (102). The enzyme Cytochrome P450 Family 51 Subfamily A Member 1 (CYP51A1) plays a crucial role in the conversion of lanosterol into meiosis-activating sterol (MAS). Lanosterol is an endogenous modulator of the innate immune function of macrophages. CYP51A1 expression was reduced in LPS/IFN-treated macrophages, which facilitated intracellular lanosterol accumulation and decreased signal transducer and activator of transcription (STAT) 1-STAT2 activation and IFN-mediated release of cytokines IL-6, TNF-α, C-C Motif chemokine ligand (CCL)-2, and INF-βin cells (103). Furthermore, the accumulation of lanosterol can promote membrane fluidity and reactive oxygen species (ROS) generation in macrophages, boosting phagocytosis and bactericidal capacity (103).

#### 3.2.4 The synthesis of zymosterol from lanosterol

Transmembrane 7 superfamily member 2 (Tm7sf2), methylsterol monooxygenase 1 (MSMO1), NAD(P) dependent steroid dehydrogenase-like (NSDHL), and hydroxysteroid 17-beta dehydrogenase 7 (HSD17B7) are four enzymes that play crucial roles in the synthesis of zymosterol from lanosterol. And the MSMO1gene is located in the psoriasis susceptibility locus PSORS9 and is a genetic risk factor for psoriasis (104). Mutations of the synthetases that synthesize zymosterol from lanosterol have the potential to impair the function of KCs. Tm7sf2 deletion impacts epidermal differentiation protein expression by lowering cholesterol sulphate levels (105, 106). Mutations in the MSMO1 gene, which encodes the sterol-C4-methyl oxidase (SMO), and the NSDHL gene can cause psoriasis-like dermatitis, inflammatory cell infiltration, and the accumulation of meiosis-activating sterol (MAS), an intermediate product of cholesterol metabolism, in the skin of patients (107, 108). Additionally, experiments done by Xiaoying Xu et al. demonstrated that HSD17B7 has a role in the regulation of KCs proliferation (109). Tm7sf2, MSMO1, and NSDHL influence inflammatory responses as well. The loss function of Tm7sf2 could activate the NF-κB pathway and up-regulate the expression of TNF-α (105, 110). Patients with SMO deficiency had a higher number of TLR-2 ^+^ TLR-4 ^-^granulocytes and increased levels of serum IL-6 and IL-8 expression, which is consistent with psoriasis patients (107). And after correction of the accumulation of intermediates, the increased IL-6 level was retracted (107). The administration of an ointment containing cholesterol and simvastatin to patients with the NSDHL gene mutation who had psoriasis-like lesions effectively restored the hyperproliferation and inflammatory process (111). Inflammatory cytokines, in turn, modulate the expression of synthetic enzymes and intermediates in cholesterol metabolism. LPS and IL-1β could enhance the expression of HSD17B7 in cells (112), and TNF-α could provoke a rise in lanosterol, MAS, zymosterol, desmosterol, and 7-dehydrocholesterol (7-DHC) in some tissues (113).

Miao He et al. proposed that MAS demethylation may be a critical link between cellular hyperproliferation, cholesterol homeostasis, and inflammation since MAS is a ligand for the liver X receptor (LXR), which is a key component linking lipid metabolism and immune regulation (107).

#### 3.2.5 Emopamil binding protein, DHCR24, DHCR7 and esmosterol

Psoriasis-like skin lesions have been observed in patients with Conradi-Hünermann-Happle syndrome, which is caused by EBP gene mutations (114). And 24-dehydrocholesterol reductase (DHCR24) knockout mice exhibit epidermal thickening, hyperproliferative hyperkeratosis with undifferentiated keratinocytes, and a considerable decrease in differentiation signal proteins in skin histology (115). Mutations in the 7-dehydrocholesterol reductase (DHCR7) gene led to an insufficiency of cholesterol, an elevation of 7-DHC and cholesta-5,7,9 (11)-trien-3beta-ol (9-DDHC) (a metabolite of 7-DHC); 9-DDHC stimulated KCs had an 88% reduction in cell viability after being explored to UVA (116). In addition to KCs, DHCR24 and DHCR7 have been demonstrated to affect the immune system by modulating the expression of their intermediate products. Desmosterol, a substrate of DHCR24, is a crucial molecule in the integration of cholesterol homeostasis and immune response in macrophages. Overexpression of DHCR24 in mice resulted in desmosterol depletion and a subsequent increase in pro-inflammatory macrophages, mitochondrial ROS, NLRP3 inflammatory vesicles, and IFN response genes, promoting vascular inflammation and atherosclerosis (117, 118). Selective DHCR24 inhibition resulted in LXR activation by raising levels of desmosterol, leading to an anti-inflammatory phenotype characterized by decreased expression of the pro-inflammatory cytokines IL-6 and TNF and increased production of the anti-inflammatory factor IL-10 (119). According to these findings, desmosterol is considered an endogenous mediator of cholesterol efflux and inflammation, acting as a primary LXR activator of atherosclerosis-associated macrophages and a negative regulator of inflammasome activation (118). However, the relevance of DHCR24 in inflammatory regulation is debatable. DHCR24 may exhibit anti-inflammatory effects by lowering vascular cell adhesion molecule 1 (VCAM-1) and NF-kB, encouraging the release of the cytoprotective and cardioprotective enzyme heme oxygenase-1 from endothelial cells, and promoting microglia to convert from a pro-inflammatory “M1” phenotype to an anti-inflammatory “M2” phenotype (120–122). It is uncertain if DHCR24’s paradoxical anti-inflammatory activity is mediated by other mechanisms. Inhibition of DHCR7 in macrophages could cause an increase in 7-DHC and a decrease in cholesterol, both of which can activate the AKT Serine/Threonine Kinase 3 (AKT3) and phosphorylation of Interferon regulatory factor 3 (IRF3) and increase production of IFN-I (123). In response to cholesterol deprivation, DHCR7 KO mast cells demonstrate FcRI-dependent hyperresponsiveness with a substantial increase in IL-6 and TNF production (124).

### 3.3 Lipoprotein and apolipoproteins

Imiquimod (IMQ)-treated psoriatic mice were paralleled by a clear dysregulation in hepatic lipid metabolism biomarkers as reflected by a decrease in HDL cholesterol and total cholesterol (125). Treatment with an anti-IL17A antibody led to a significant reversal of IMQ-induced changes in the liver biomarkers of lipid metabolism, whereas treatment with IL-17A further aggravated IMQ-induced changes in these biomarkers (125). Another study also showed that serum total cholesterol, and triglyceride in the high-fat-feed rats decreased after being injected with the IL-17 neutralizing antibody (126). LDL peroxidation results in oxidized low-density lipoprotein, often known as “ oxidized low-density lipoprotein (ox-LDL).” Ox-LDL is taken up by transmembrane scavenger receptors like SR-B1 and CD36, as well as by the lectin-like oxidized LDL receptor-1 (LOX-1) (127, 128). Excess oxygen metabolites overwhelm the antioxidant capacity of the organism, which is one of the important features of psoriasis (129). And the value of LDL in psoriasis is inconsistent and ambiguous; hence, ox-LDL, rather than LDL, is thought to be more meaningful to psoriasis (130). Consistent with this idea, psoriasis patients exhibit higher epidermal and serum ox-LDL levels, as well as autoantibodies against ox-LDL (15). Also, lipid peroxidation markers were associated with the severity and activity of psoriasis (129). Ox-LDL can engage in the progression of psoriasis through multiple mechanisms. On the one hand, ox-LDL could promote the production of IL-23 in dendritic cells (DCs), which is a key cytokine in the pathophysiology of psoriasis (131). A study by Chun-Ming Shih et al. also found that ox-LDL boosted IL-23 expression in TNF-α stimulated KCs *via* LOX-1, which is elevated and linked with the severity of the disease in psoriatic patients (127, 132). On the other hand, by over-expressing CD36 to uptake ox-LDL and form foam cells, psoriatic monocytes are more likely to evolve into M1 pro-inflammatory macrophages that produce psoriatic cytokines and raise the risk of cardiovascular disease comorbidity (128). In turn, pro-inflammatory cytokines such as TNF-α, IL-1β, and IL-6, which are increased in psoriasis, can activate LOX-1, promoting the uptake of ox-LDL by macrophages (133).

HDL takes part in reverse cholesterol transport (RCT) and possesses anti-inflammatory and antioxidant effects. HDL is responsible for the one-way transport of cholesterol to either apolipoprotein (apo) A-I *via* ABCA1, mature HDL particles *via* ABCG1, or the liver for metabolism by SR-B1 (134). By modulating the differentiation and function of immune cells such as macrophages, DCs, T cells, and B cells *in vivo*, HDL and apoA-1 can achieve an anti-inflammatory effect (135). Correspondingly, inflammation also drops the level of HDL, although the mechanism is still unknown. But it is believed that the decreased levels of HDL-associated proteins apoA-1, apoM, lecithin: cholesterol acyl transfer (LCAT), and CETP may all contribute (136). Besides, inflammation also impairs HDL’s capability to engage in RCT and protect LDL cholesterol from oxidation (136). In psoriasis, HDL cholesterol levels were decreased compared to healthy controls (15). Furthermore, the composition and function of HDL are altered in psoriasis, resulting in a decrease in cholesterol outflow and an impairment of anti-inflammatory and anti-oxidation of HDL (137). The expression of HDL-related proteins apoA-1, apoM, and apoF is reduced in psoriasis, whereas apoA-2, which plays a negative regulatory role, is increased (137). The activity of LACT, which is an important enzyme in the formation of HDL particles, was decreased in psoriasis patients, while its activity increased after biologics and other anti-psoriatic treatments in psoriasis patients (138). However, the change in HDL in psoriatic patients with anti-psoriatic medication appears paradoxical (27). Overall, HDL cholesterol efflux capability was impaired in psoriasis patients and was negatively correlated with the severity of the condition and the risk of cardiovascular comorbidities (27). As a result, anti-psoriatic medication focusing on HDL may benefit psoriatic patients suffering from cardiovascular issues.

Apolipoprotein E (apo E) is a multifunctional glycosylated protein with lipid transport immunomodulatory properties (139). Apo E shows a function of anti-inflammation and could intersect with inflammatory factors. Apo E promotes the transition of pro-inflammatory M1 macrophages to anti-inflammatory M2 macrophages (139). Compared to wild-type mice, mice that lack Apo E displayed a higher level of TNF-αand a higher mortality rate in response to the infection of pathogens (140). Additionally, Apo E deficiency may result in an increase in pro-inflammatory factors(IL-1, IL-2, IL-6, IFN-γand ICAM-1) and a decrease in anti-inflammatory factors (IL-4 and IL-10), promoting a Th1 immune response and an imbalance between Th17 cells and Regulatory T cells (Tregs) (139). Correspondingly, IFN-γ, TNF-α, and granulocyte-macrophage colony-stimulating factor (GM-CSF)-stimulated macrophages had a reduction of apoE (139). ApoE expression is reduced in psoriatic skin than in healthy controls, and restoration of apoE levels precedes clinical improvement (141). Furthermore, ApoE gene polymorphisms are correlated with psoriasis, indicating that ApoE may have a pathogenic role in psoriasis (142).

### 3.4 Lipoprotein receptors and metabolism

Previous biochemical and morphologic studies have revealed that LDL receptors (LDLR) activity is inversely related to the terminal differentiation of KCs (105). LDL receptor binding activity is only found in the basal cells and not in highly differentiated KCs in the normal epidermis (143). However, differentiated acanthocytes have higher LDLR activity in psoriasis (143). Similarly, LRP1 levels were higher in psoriasis patients, but anti-inflammatory molecules LRP5 and LRP6 were lower in psoriatic lesions and peripheral blood (144, 145).

Proprotein convertase subtilisin/kexin type 9 (PCSK9), a target for decreasing LDL in the blood, sits at the crossroads of cholesterol metabolism and immune dysregulation. PCSK9 interactives with inflammation. PCSK9 regulates atherosclerotic plaque and inflammation in both lipid-dependent and lipid-independent ways, and its pro-inflammatory effect depends on two major signaling pathways: the principal pathway through which PCSK9 acts is the TLR4/NF-kB signaling pathway, while the LOX-1 axis is another linked pathway (146). PCSK9 activates NF-kB, which in turns activates TLR4 and LOX-1to generate TNF-α, IL-6, IL-1, and macrophage chemoattractant protein 1 (MCP-1) (146). In contrast, TNF-α and IL-6 can also stimulate the expression of PCSK9 (147). Recent studies showed that PCSK9 takes part in the development of psoriasis. Luan et al. described a direct link between PCSK9 and psoriasis for the first time (148). Their research found that psoriasis patients and IMQ-treated mice both had elevated PCSK9, and that inhibiting PCSK9 could lessen the inflammatory response in psoriasis by inhibiting KCs proliferation and the NF-κB pathway (148). According to their research, targeting PCSK9 may be a promising therapeutic for psoriasis. Garshick et al. further found that the level of PCSK9 was higher in psoriasis patients and K14-Rac1V12 -/+ psoriatic mice and was positively linked with the severity of psoriasis (149). Furthermore, they postulated that PCSK9 is connected with a higher atherosclerotic burden in psoriasis patients, and, using blood transcript analysis, they identified TNF, IL6, IL17A, and IL23 as PCSK9-related pathways (149). Another recently published study found that the PCSK9 single nucleotide polymorphism (SNP) rs662145 was a psoriasis susceptibility locus and that PCSK9 expression is negatively linked to IL-36, a critical driver of psoriasis (150). These findings will aid future studies into PCSK9’s role and mechanism in psoriasis. Only one clinical study explored the therapeutic effect of PCSK9 inhibitors (e.g., alirocumab) on psoriasis, and the results showed that inhibition of PCSK9 was associated with a reduced psoriasis risk (43).

### 3.5 CETP, ACAT

CETP is a lipid transport protein in plasma that moves CEs from HDL to lipoproteins with apoB (151). CETP may have contradictory roles in inflammation. CETP gene gain-of-function mutations are associated with elevated IL-8 and decreased plasma HDL cholesterol levels (152). After LPS stimulation, macrophages from mice overexpressing CETP produced greater TNF-α, IL-1, IL-6, MCP-1, and IFN-γ, accompanied by a lower level of total and esterified cholesterol and a higher sepsis mortality rate (153). Nevertheless, several studies have shown that CEPT may have an anti-inflammatory role. CEPT may prevent macrophages with the M1 phenotype from switching to the M2 phenotype. CEPT also decreases the production of TNF-α, IL-6, and iNOS. And it also lowers the levels of free cholesterol and cell phagocytosis (144, 153, 154). Jun Chen et al. investigated the role of CETP in psoriasis. And the results showed that CETP-transgenic mice had significantly higher levels of IFN-γ, IL-1, IL-6, IL-17A, IL-17F, IL-22, and IL-23 in lesions and higher serum levels of TNF-α, IL-17, and IL-6 after IMQ-stimulation (151). The results of the study by Jun Chen et al. implied that CETP could aggravate psoriasis, and this could be CETP’s role in regulating inflammation and blood lipids. Then, we searched for studies that evaluated serum CETP concentrations in psoriasis patients. And the results showed that the expression of CETP was different. It was lower in some psoriatic patients, higher in others, and no difference in still others (155).

ACAT, also known as sterol O-acyltransferase (SOAT), is a therapeutic target for atherosclerosis; by converting cholesterol to CEs, ACAT is essential for maintaining cellular cholesterol homeostasis (156). ACAT1 is expressed by almost every cell in the body, whereas ACAT2 is mostly expressed by the liver and intestines but also by other tissues (156). ACAT has not yet been researched in psoriasis, but a series of studies show that ACAT has an interaction with immune cells and cytokines that exert an important role in psoriasis. ACAT1 can reduce cell proliferation and induce cell apoptosis (157). A bioinformatics study showed that the expression of ACAT1 was positively linked to macrophages, neutrophils, Th17 cells, and activated dendritic cells but negatively linked to plasmacytoid dendritic cells or natural killer cells (158). Inhibiting ACAT1 decreases the number of inflammatory monocytes and macrophages and changes them to an anti-inflammatory M2 phenotype, and blocking ACTA1 also decreases the expression of inflammatory factors like MCP-1 and CCL5/7 (159). Blocking ACAT1 with avasimibe significantly reduced serum TNF-αlevel in hypercholesterolemic patients (160). In turn, in monocytes and macrophages, IFN-γ, IL-1, TNF-α, and LPS can increase the expression of ACAT1 and promote the formation of CE-laden cells, and these effects are mostly dependent on the NF-B pathway (161, 162). ACAT inhibition is speculated to achieve its anti-inflammatory effect through increasing free cholesterol levels or altering oxysterol levels (156).

### 3.6 ATP-binding cassette transporters

ABCG1 and ABCA1 are transport proteins that are related to RCT and have been used as therapeutic targets to lower lipids. ABCG1 was involved in epidermal differentiation. It was induced during KCs differentiation and was highly expressed in the outer epidermis (163). A series of studies have revealed that ABCG1- and ABCA1-mediated lipid transport has potent anti-inflammatory capabilities. ABCA1 and ABCG1 suppress innate immunological responses by reducing cholesterol-rich lipid raft membrane microdomains (164). ABCA1 preferentially lowered the FC concentration in lipid rafts and inhibited MyD88-dependent TLR translocation to lipid rafts, further reducing the inflammation in macrophages (165). Co-knockdown of ABCG1 and ABCA1 in DCs results in lipid buildup in DCs, which in turn leads to inflammasome activation, an increase of IL-1 and IL-18, and a phenotypic shift of T cells toward a Th1 phenotype (165). Moreover, the DC-ABCA1/G1 deficit increased GM-CSF-induced production of IL-23, IL-12, IL-17, and IL-6, as well as the differentiation of T cells into TH17 cells in splenic CD11c+ DCs (165). Inversely, inflammatory cytokines such as IL-17A, TNF, IL-1, and IL-10 can impact the expression levels of ABCA1 and ABCG1 *in vivo (* 166–168). According to a review by John M. Gemery et al., ABCA1^-/-^ and ABCG1-/- mice have chronic stem cell mobilization, accelerated atherosclerosis, and increased IL-17, IL-23, and granulocyte colony-stimulating factor (G-CSF); in addition, elevated IL-17 and IL-23 in psoriasis might also induce stem cell upregulation/mobilization, which could hasten the establishment of atherosclerosis (169, 170). Hence, John M. Gemery et al. proposed that ABCA1 and ABCG1 may play a role in psoriasis *via* regulating inflammation, cholesterol metabolism, and cardiovascular complications. It is plausible to question if psoriasis-related inflammatory factors can interact with ABCG1 or ABCA1 to disrupt cholesterol metabolism and epidermal differentiation and exacerbate the inflammatory response.

ABCG5/8 is mostly expressed in the gut and liver, and it regulates TLR-induced inflammation (171). Additionally, inflammation adversely influences ABCG5/8 expression to impede cholesterol transport from the liver to the bile (172). Mice injected with IL-1β exhibited a time-dependent reduction in hepatic ABCG5 expression (173). Given a bile acid imbalance has been observed in patients with psoriasis and psoriatic arthritis (174) and the role of ABCG5/8 in the TICE, we speculate whether ABCG5 and ABCG8 play a role in the bile acid metabolism of psoriasis.

## 4 Phytosterols and psoriasis

Phytosterols are naturally active chemicals present in many plants (especially in whole grains, vegetable oils, nuts, seeds, and legumes) that has a structure similar to cholesterol (175). Phytosterols are classified into sterols (primary products) and stanols based on their chemical structure (175). Over 250 phytosterols have been identified, with the main types including brassicasterol, campesterol, stigmasterol, and β-sitosterol (175). Phytosterols compete with cholesterol to produce micelles, which are then absorbed in the gut *via* NPC1L1 (176). Esterified phytosterols are incorporated into chylomicrons and then absorbed by the liver, whereas unesterified phytosterols are excreted to the intestinal cavity by ATP -binding cassette transporters (175).

A series of preclinical and clinical studies have shown that phytosterols may confer various bioactive effects, including anticancer, antioxidant, anti-inflammatory, antidiabetic, effects on lipid profile, and neuroactive effects (177). On the basis of the dysregulation of cholesterol metabolism in psoriasis and the lipid-lowering impact of phytosterols, the potential function of phytosterols in psoriasis was discussed.

The dichloromethane extracts of Melissa officinalis ssp. Altissima include campesterol, and the dichloromethane extracts have an anti-psoriatic activity to improve the clinical and pathological symptoms (178). Withasteroids are a group of steroidal compounds extracted from Datura metel L and in which withanolides are the most effective part for psoriatic treatment (179).

The same as with sitosterol, treatment with withanolides significantly improved the clinical symptoms and pathological manifestations (epidermal hyperplasia, acanthosis, hyperkeratosis, and abundant inflammatory infiltrate) and decreased the PASI score in IMQ-induced psoriatic mice (180, 181). Withanolides exert their antipsoriatic effects through various mechanisms. Withanolides can directly affect the biofunction of KC. Withanolides could induce the expression of autophagy factors, initiate the senescence and apoptosis of KCs, and inhibit KCs’ proliferation and migration induced by IL-17 (182, 183). Additionally, withanolides inhibited janus kinase (JAK)/STAT-dependent CD4+ T cell differentiation with suppression of Th17 cells and an increase in the Tregs cell type (179). And, withanolides and sitosterol significantly reversed the elevated IL-10, IL-17, IL-22, IL-23, IL-6, TNF-α, IL-8 and CXCL1 in psoriatic models (180, 181). The anti-inflammation function of withanolides may partially rely on the inhibition of the NF-κB pathway (183).

## 5 Conclusion

Psoriasis has been shown to have a negative physical, psychological, and social impact on patients, and the accumulated effects may lead to patients being unable to reach their “full life potential”, which is referred to as “cumulative life course disorder” (184). Additionally, psoriasis imposes a significant financial burden, with patients paying $11,498 over their lifetime for the relief of clinical symptoms and emotional well-being and the United States paying approximately $112 billion for psoriasis in 2013 (185). And since systemic comorbidities, especially cardiovascular disorders, are very common in psoriatic patients, it is essential to develop new targets for the treatment of psoriatic patients with different comorbidities.

The imbalance of cholesterol metabolism contributes significantly to the pathogenesis of psoriasis. Disorders of cholesterol metabolism, such as an increase of serum LDL cholesterol and VLDL cholesterol and a decrease of serum HDL cholesterol, can be observed in patients with psoriasis. Alterations in molecules related to cholesterol metabolism are proven to be correlated with psoriasis-related comorbidities such as cardiovascular disease, obesity, and hyperlipidemia. In recent years, metabolomics and immunometabolism have become popular research topics. Numerous molecules engaged in cholesterol metabolism are responsible for the regulation of immunological disorders and inflammatory responses in physiology and pathology. Moreover, chronic inflammation can influence the lipid profiles of diseases. However, current research on cholesterol metabolism in psoriasis is restricted to serum lipid, apo, and lipid receptors. The research on intermediate products and bile acid metabolites formed during cholesterol metabolism in psoriasis is still sparse.

In our review, we discussed the relationship between various molecules involved in cholesterol metabolism and key cytokines involved in psoriatic inflammation. Our review may provide new insights for further research between immunometabolism disturbance and psoriasis and provide new targets for the therapy for psoriasis and its complications.

## Author contributions

Conceptualization, CL, LL and YG. Funding acquisition, CL. Writing – Original Draft Preparation, LL. Writing – Review & Editing, LC, JZ, YG, and CL. All authors contributed to the article and approved the submitted version.
